# Laparoscopic Right Hemicolectomy in an Automated Peritoneal Dialysis Patient without Removal of the PD Catheter: A Case Report

**DOI:** 10.1155/2014/492567

**Published:** 2014-07-08

**Authors:** Joseph A. Attard, Alexander Attard

**Affiliations:** Department of Surgery, St. James's Hospital, Borg Olivier Street, Sliema SLM 1807, Malta

## Abstract

*Introduction*. Laparotomy in patients on peritoneal dialysis (PD) is associated with an increased risk of morbidity. Furthermore, standard protocol recommends removal of the PD catheter when surgery on the intestine is required. As far as we are aware, this is the first case report of laparoscopic right hemicolectomy in a patient on automated PD where the PD catheter was left in situ. *Case Report*. A 61-year-old man man on APD who presented with a caecal carcinoma was stabilised on temporary haemodialysis (HD) prior to undergoing a laparoscopic right hemicolectomy without removal of the PD catheter. He made an uneventful recovery and APD was resumed successfully 2 weeks after surgery. *Discussion*. PD patients undergoing intra-abdominal surgery are at increased risk of complications. While the benefits of laparoscopic surgery in the standard surgical population are well established, there is limited experience of the technique in PD patients. Possible advantages could theoretically be early resumption of PD as well as less PD failure due to the formation of adhesions. *Conclusion.* Our experience with this case indicates that laparoscopic right hemicolectomy in a background of PD can be undertaken without removal of the PD catheter and is associated with early resumption of PD.

## 1. Introduction

Peritoneal dialysis gives patients the choice of an effective home modality of renal replacement therapy for end stage renal disease and represents 40% of patients on dialysis in our institution. However, open surgery on the abdomen may lead to complications such as failure of catheter insertion, failure of resumption of dialysis after surgery, poor wound healing, higher rates of wound infection, and leakage of dialysate fluid [1]. The benefits, if any, of adopting the laparoscopic approach in these patients have not yet been determined. While certain relatively “clean” laparoscopic procedures have been reported in patients on PD without removal of the PD catheter, the general recommendation is that the PD catheter is removed following any procedure performed in a potentially contaminated environment. We present our experience in a PD patient diagnosed with a caecal carcinoma who successfully underwent laparoscopic right hemicolectomy without removal of the PD catheter.

## 2. Case Report

Our patient is a 61-year-old man who had been doing well on APD for 4 years and whose haemoglobin was being maintained by regular injections of erythropoietin. Routine haemoglobin assay, initially normal, suggested the development of iron deficiency anaemia and he was therefore referred for colonoscopy. This showed the presence of a caecal carcinoma. Staging CT did not reveal any spread. He was established on haemodialysis (HD) via a temporary venous line and underwent laparoscopic right hemicolectomy through 4 ports. Pneumoperitoneum was established via the PD catheter which was left in situ. Extracorporeal anastomosis was achieved using GIA and TA stapling devices (Covidien, Mansfield, USA) through slight extension of the right subcostal port incision. Cefuroxime and metronidazole were used to cover the procedure preoperatively and for 48 hours after surgery. The catheter was flushed daily with heparinised saline until resumption of APD and the effluent was sent for culture. Bowel sounds returned on the second postoperative day and he was on a normal diet by the third day after his surgery. He was discharged on the fifth postoperative day and APD was resumed 2 weeks after his surgery. None of the effluent cultures were positive for bacterial growth. Histology showed Duke's A carcinoma of the caecum. He remained well, free of tumour recurrence, and on APD until his death from myocardial infarction 3 years later.

## 3. Discussion

Renal transplantation remains the preferred treatment for patients with end stage renal disease (ESRD) [2]. However, most patients with ESRD are put on some form of dialysis while awaiting transplantation and, in some cases, permanently. While HD was the standard treatment in these patients for many years, PD became increasingly popular following the development of the first permanent PD catheter by Tenckhoff and Schechter in 1968 [3]. Recent reports show that PD, while being a less expensive method of renal replacement therapy when compared to HD, is not associated with increased mortality [4].

Previous abdominal surgery on patients earmarked for PD can have a significant impact on the success of this treatment due to the presence of adhesions which may prevent successful insertion of the PD catheter or cause catheter malfunction after insertion. A previous history of abdominal surgery is the commonest reason for patients not being considered for PD [5]. The adoption of laparoscopic techniques instead of open surgery may hopefully reduce this problem but experience in these patients is limited. Furthermore, patients already on PD undergoing laparotomy have a higher risk of complications. These include poor wound healing, wound infection, leakage of dialysate fluid through the wound, and catheter failure due to infection or adhesion formation [1]. In view of this, PD catheters are almost invariably removed following laparotomy in a potentially contaminated environment. Some authors also report a high incidence (71%) of peritonitis in PD patients undergoing laparoscopic procedures in a relatively clean environment and suggest that this can be eliminated by temporary cessation of PD for 2 weeks after surgery [6]. Recent reports suggest that it is possible to successfully and safely perform laparoscopic procedures in patients on PD without removing the PD catheter and with a relatively short period of HD in the interim period before resuming PD. Laparoscopic cholecystectomy [7–9], laparoscopic radical nephrectomy [10], and laparoscopic appendicectomy for perforated appendicitis [11] have been reported using this approach. It is interesting that, even though the latter procedure was carried out in an infected environment, PD was resumed successfully and no infective complications were reported. Our own experience, as described above, indicates that, despite potentially contaminating the operative field and peritoneal cavity during the course of anastomosing the bowel and replacing the possibly contaminated stapled anastomotic line back into the abdomen, no infective complications were encountered nor were there any bacteria cultured from the catheter effluent taken on a daily basis until resumption of APD 2 weeks after surgery.

A 5-year retrospective review of PD patients undergoing laparotomy in our institution revealed that none of these patients were referred for reinsertion of the PD catheter for eventual resumption of PD on the assumption that this would fail for the reasons outlined previously. In this case, the laparoscopic approach allowed for faster recovery and discharge of the patient and early successful resumption of APD after discharge, up to his death from an unrelated cause 3 years later.

It is too early to assess whether the laparoscopic approach for colonic surgery in PD patients is as safe as or superior to the open technique since this is, as far as we are aware, the first and only case of laparoscopic right hemicolectomy without removal of the PD catheter reported. Torigoe et al. reported a case of laparoscopic transverse colectomy, leaving the PD catheter in situ [12]. In an effort to ensure a successful outcome we have developed a protocol to be used in such patients based on our experience with the case reported here. This is presented as follows.

*Protocol for Use in Laparoscopic GI Surgery in PD Patients.*

- Temporary HD to be established prior to surgery.
- PD catheter to be left in situ.
- Broad spectrum antibiotics at induction and for 48 hours after surgery.
- Daily flushes of PD catheter with heparinised saline from day 1 post-op until resumption of PD.
- Daily culture of effluent and close monitoring for possible signs of sepsis until resumption of PD.
- Resumption of PD 2 weeks after surgery [6].

The safety of laparoscopic colectomy without removal of the PD catheter in such patients can only be determined with more experience and hopefully other centres will report on their experiences in the future. In the case presented here, no complications were observed and we propose that the protocol suggested could be considered for use in such cases to hopefully reduce the risks of morbidity and mortality in these patients.
